# Identification of Common Angiogenesis Marker Genes in Chronic Lung Diseases and Their Relationship with Immune Infiltration Based on Bioinformatics Approaches

**DOI:** 10.3390/biomedicines13020331

**Published:** 2025-01-31

**Authors:** Lu Liu, Man Wang, Shihuan Yu

**Affiliations:** Department of Respiratory Medicine, the First Affiliated Hospital of Harbin Medical University, Harbin 150001, China; ivyliu1991@126.com (L.L.); wmpost@sina.cn (M.W.)

**Keywords:** chronic lung diseases, angiogenesis-related genes, bioinformatics methods, key gene screening, therapeutic targets

## Abstract

**Objective:** This study aims to explore the role of angiogenesis-related genes in chronic lung diseases (ILD and COPD) using bioinformatics methods, with the goal of identifying novel therapeutic targets to slow disease progression and prevent its deterioration into fibrosis or pulmonary artery hypertension. **Methods:** The research methods encompassed differential analysis, WGCNA (Weighted Gene Co-expression Network Analysis), and multiple machine learning approaches to screen for key genes. Gene Set Enrichment Analysis (GSEA), Gene Ontology (GO), and the Kyoto Encyclopedia of Genes and Genomes (KEGG) were utilized to assess related biological functions and pathways. Additionally, immune cell infiltration was analyzed to evaluate the immune status of the disease and the correlation between genes and immunity. **Results:** COPD and ILD are closely associated with pathways related to angiogenesis, immune responses, and others, with differential genes in both groups linked to inflammation-related signaling pathways. The study established a chronic lung disease-related gene set comprising 171 genes and further screened out 21 genes related to angiogenesis. Ultimately, four key genes—*COL10A1*, *EDN1*, *MMP1*, and *RRAS*—were identified through machine learning methods. These four genes are closely related to angiogenesis and immune processes, and clustering analysis based on them can reflect different disease states and variations in immune cell infiltration. **Conclusions:** *COL10A1*, *EDN1*, *MMP1*, and *RRAS* represent potential therapeutic targets for slowing the progression of chronic lung diseases and preventing their deterioration. Furthermore, monocytes exhibited consistent infiltration patterns across disease and control groups, as well as among different subgroups, suggesting their potential significant role in the development of chronic lung diseases.

## 1. Introduction

Chronic lung diseases represent one of the leading causes of disability and mortality worldwide. Currently, chronic obstructive pulmonary disease (COPD) tops the list of chronic lung diseases in terms of global prevalence for both males and females, with an absolute upward trend in its incidence [1]. Chronic lung diseases primarily encompass COPD, interstitial lung disease (ILD), and pulmonary artery hypertension (PAH), among others, wherein pathogenesis involves processes of lung regeneration and remodeling, particularly changes in the pulmonary vascular system. Maintaining an intact vascular structure is crucial for preserving a functional pulmonary blood–gas barrier. The development and remodeling of the pulmonary vascular system constitute a complex morphogenetic process, including the formation of new blood vessels and the matching of ventilation and perfusion [2]. Angiogenesis, a multistep process whereby new capillaries form from pre-existing vessels, is tightly regulated by the balance of various modulators within the vascular microenvironment. Pathological angiogenesis is associated with a range of diseases, including cancer, diabetic retinopathy, autoimmune disorders, and more [3].

Angiogenesis is a complex and intricate process involving the expression and regulation of multiple angiogenesis-related genes (ARGs). ARGs refer to those that encode proteins or factors that regulate the angiogenic process, with their expression levels and mutations directly influencing the speed, direction, and pattern of angiogenesis. Under normal circumstances, the expression of these genes is tightly regulated to ensure the proper progression of angiogenesis. However, in pathological conditions, such as chronic lung diseases, abnormalities may occur in the expression and regulation of these genes, leading to disruptions in the angiogenic process and subsequently promoting the onset and progression of the disease.

Indeed, the role of angiogenesis in chronic lung diseases has been extensively studied. In chronic lung diseases, both inhibition of angiogenesis and excessive angiogenesis can disrupt angiogenic homeostasis, leading to abnormal lung repair, structural disarray, and promoting the development of pulmonary artery hypertension (PAH) and fibrosis, thereby exacerbating the disease condition. For example, interstitial lung disease (ILD) caused by different etiologies may exhibit distinct angiogenic states. Nonspecific interstitial pneumonia (NSIP) and acute fibrinous and organizing pneumonia (AFE) primarily manifest as significant vascular remodeling in thickened alveolar septa, whereas the lung characteristics of usual interstitial pneumonia (UIP) include increased upstream vessel density and reduced vessel density in perilesional and peri-alveolar capillary plexuses [4,5]. Reducing the production of inflammatory mediators through immunomodulatory effects can promote angiogenesis to increase blood supply to damaged areas, facilitating tissue repair and regeneration [6,7,8]. In the pathological process of COPD, various factors such as inflammation and hypoxia can induce the expression of angiogenesis-related factors, including vascular endothelial growth factor (VEGF), thereby promoting pulmonary vascular remodeling and the formation of new blood vessels [9]. Angiogenesis is a hallmark of airway inflammation in COPD, and inflammatory effector cells are a major source of numerous angiogenic factors [9]. VEGF is the most potent activator of angiogenesis, stimulating the migration and proliferation of endothelial cells to generate and stabilize new blood vessels, leading to bronchial cell remodeling and inflammation, which in turn contribute to the onset and progression of various pulmonary diseases including PAH, COPD, bronchial asthma, and ILD [10]. Meanwhile, pulmonary vascular remodeling and the formation of new blood vessels are central components of PAH [11,12]. In PAH, changes in hemodynamic forces affect pulmonary endothelial cell function, leading to immune cell adhesion and the release of inflammatory mediators. The resulting perivascular inflammation, in turn, promotes vascular remodeling and the progression of PAH. This vicious cycle of endothelial activation, inflammation, and vascular remodeling may drive the onset and progression of the disease [13].

Therefore, this study intends to explore the shared angiogenesis-related genes in chronic lung diseases (ILD and COPD) and their relationship with immune infiltration through bioinformatics methods. The aim is to gain a deeper understanding of the role of angiogenesis in chronic lung diseases and how these roles interact with immunity and inflammation, thereby limiting the onset and progression of chronic lung diseases. It is hoped that through this study, new therapeutic targets can be identified to slow down the progression of chronic lung diseases and prevent their deterioration towards fibrosis and pulmonary artery hypertension.

## 2. Methods

### 2.1. Data Source and Processing

The Gene Expression Omnibus (https://www.ncbi.nlm.nih.gov/geo/ (accessed on 10 November 2024)) database from the National Center for Biotechnology Information (NCBI) was searched. The GSE47460 dataset was selected, which comprises human lung tissue samples from 254 patients with ILD, 220 patients with COPD, and 108 from the control (CTRL) group. The dataset is divided into two platforms, GPL6480 and GPL14550. The R package inSilicoMerging was utilized to merge the dataset [14], and no significant batch effect was observed.

### 2.2. Differential Analysis and Functional Enrichment Analysis

Differential expression analysis was performed using the limma package (v3.40.6) in R [15], with selection criteria set at a fold Change greater than 1.5 and a *p*-value less than 0.05. The clusterProfiler package (v3.14.3) was utilized for Gene Ontology (GO) and Kyoto Encyclopedia of Genes and Genomes (KEGG) enrichment analysis of the differentially expressed genes. For Gene Set Enrichment Analysis (GSEA), the GSEA software (v3.0) and gene sets from the Molecular Signatures Database (MSigDB) were employed to evaluate the relationship between gene expression profiles and specific biological functions or pathways.

### 2.3. Weighted Gene Co-Expression Network Analysis (WGCNA)

The data were preprocessed, and outliers were removed by calculating the Median Absolute Deviation (MAD) values and applying the goodSamplesGenes function from the R package. A scale-free co-expression network was constructed, which involved calculating the Pearson correlation coefficient matrix, building a weighted adjacency matrix, transforming it into a Topological Overlap Matrix (TOM), and performing average linkage hierarchical clustering based on this to establish co-expression modules. Modules significantly associated with the disease group were selected, and core genes were screened using thresholds of |MM| > 0.8 and |GS| > 0.1 [16].

### 2.4. Machine Learning for Feature Gene Selection

In this study, three machine learning algorithms were employed to select feature genes, including Least Absolute Shrinkage and Selection Operator (LASSO) logistic regression, Random Forest (RF), and Support Vector Machine Recursive Feature Elimination (SVM-RFE) [17,18,19,20]. These algorithms are of great significance for identifying key biomarkers. Currently, these three algorithms are widely used in research to identify key genes [21,22,23]. Potential biomarkers were determined based on the feature genes identified through the cross-validation of LASSO, RF, and SVM-RFE algorithms.

### 2.5. Immune Infiltration Analysis

Immune infiltration analysis was conducted using ImmuCellAI (https://guolab.wchscu.cn/ (accessed on 10 November 2024)) [24]. ImmuCellAI can assess the abundance of 24 types of immune cells in human samples, including 18 types of T cells and 6 other immune cell types.

### 2.6. Clustering Analysis

Clustering analysis was performed using ConsensusClusterPlus [25]. Specifically, the agglomerative PAM (Partitioning Around Medoids) clustering algorithm based on 1-Pearson correlation distance was employed, and a resampling strategy was used to select 80% of the samples for 10 independent replicate experiments. The optimal number of clusters was determined through the empirical cumulative distribution function plot.

### 2.7. Statistical Analysis

Data were analyzed using R software (v.4.4.1) and the SangerBox 3.0 [26] online platform. The Wilcoxon rank-sum test and t-test were employed for inter-group difference analysis. A *p*-value < 0.05 was considered statistically significant.

## 3. Results

### 3.1. GSEA of COPD and ILD

**Figure 1:**
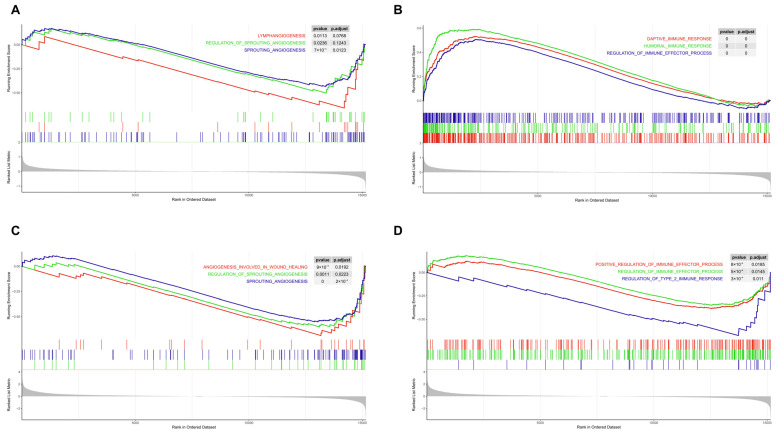
GSEA Based on the GO Database. (**A**): The relationship between COPD and angiogenesis-related pathways; (**B**) the relationship between COPD and immune-related pathways; (**C**) the relationship between ILD and angiogenesis-related pathways; (**D**) the relationship between ILD and immune-related pathways.

Functional GSEA in the COPD and ILD groups revealed that both COPD and ILD are closely associated with pathways related to angiogenesis, regulation of angiogenesis and neovascularization, and regulation of immune processes. These findings suggest that imbalances in angiogenic homeostasis and dysfunction of the immune system may be core mechanisms underlying the development and progression of chronic lung diseases (COPD and ILD) (Figure 1).

### 3.2. Identification of Differentially Expressed Genes

**Figure 2:**
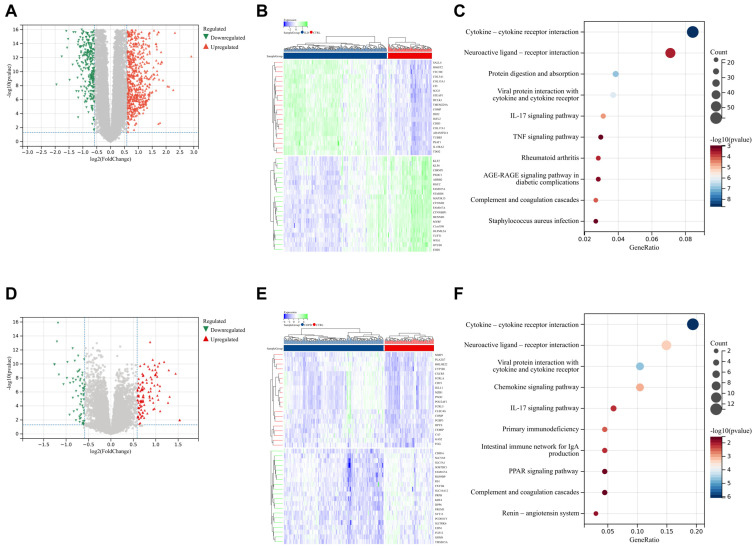
Differential Analysis Using limma and the KEGG. (**A**) Volcano plot of DEGs between the ILD and CTRL groups; (**B**) heatmap showing the top 20 upregulated and downregulated DEGs between the ILD and CTRL groups; (**C**) KEEG analysis of DEGs between the ILD and CTRL groups. (**D**) volcano plot of DEGs between the COPD and CTRL groups; (**E**) heatmap showing the top 20 upregulated and downregulated DEGs between the COPD and CTRL groups; (**F**) KEEG analysis of DEGs between the COPD and CTRL groups.

Further differential expression analysis and KEGG gene enrichment analysis were conducted. Compared to the CTRL group, the ILD group identified 975 upregulated genes and 735 downregulated genes. KEGG analysis indicated that these differentially expressed genes (DEGs) were primarily enriched in several key pathways, including cytokine–cytokine receptor interactions, the IL-17 signaling pathway, and the TNF signaling pathway (Figure 2A–C). When comparing the COPD group to the CTRL group, 110 upregulated genes and 64 downregulated genes were identified. KEGG analysis showed that these genes were also significantly enriched in pathways such as cytokine–cytokine receptor interactions, the IL-17 signaling pathway, and the chemokine signaling pathway (Figure 2D–F). Despite differences in the number and specific enrichment levels of differentially expressed genes between ILD and COPD groups, both exhibited similar enrichment patterns. These results not only emphasize the central role of inflammation in the pathogenesis of chronic lung diseases but also further confirm the molecular-level commonalities between ILD and COPD.

### 3.3. WGCNA

**Figure 3:**
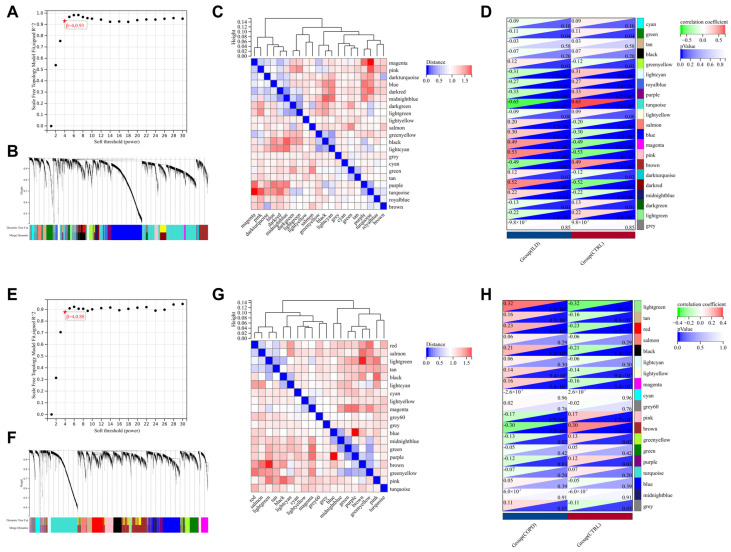
Further Screening of Gene Sets Highly Relevant to ILD and COPD through WGCNA. (**A**–**D**) represent the WGCNA for the ILD group. (**A**) Selection of an appropriate soft threshold; (**B**,**C**) generation of 21 modules; (**D**) correlation of modules with ILD. (**E**–**H**) represent the WGCNA for the COPD group. (**E**) Selection of an appropriate soft threshold; (**F**,**G**) generation of 19 modules; (**H**) correlation of modules with COPD.

To further screen for gene sets highly correlated with the ILD and COPD groups, we performed WGCNA on the dataset. In the ILD patient group, the soft thresholding power was calibrated to 4 (scale-free R^2^ = 0.93), and a total of 21 modules were identified, among which the turquoise, pink, and darkred modules showed the most significant association with ILD. From these three modules, 386 key genes were further screened (Figure 3A–D). In the COPD patient group, the soft thresholding power was calibrated to 4 (scale-free R^2^ = 0.88), and a total of 19 modules were determined, with the lightgreen and brown modules showing the closest association with COPD. From these two modules, 93 key genes were screened (Figure 3E–H).

### 3.4. Identification of Angiogenesis-Related Genes Associated with Chronic Lung Diseases

**Figure 4:**
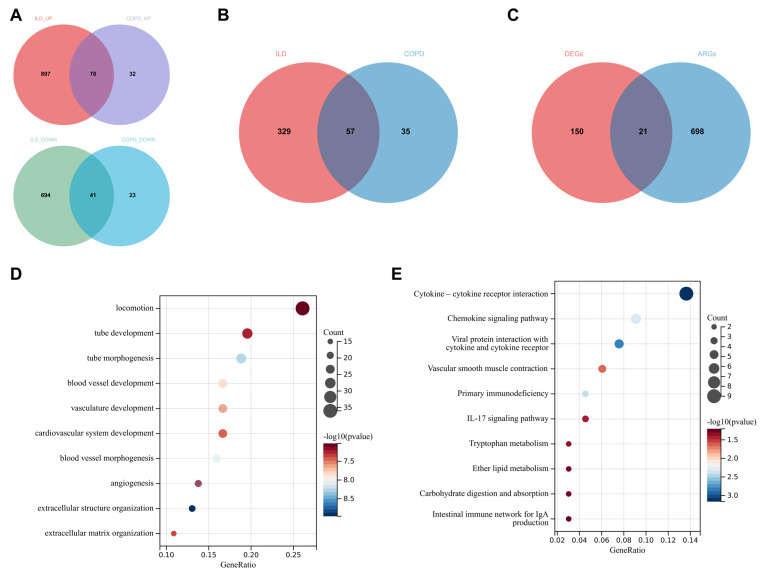
Screening of Angiogenesis-Related Genes Associated with Chronic Lung Diseases. (**A**) There are 78 upregulated and 41 downregulated common differentially expressed genes (DEGs) in both the ILD and COPD groups; (**B**) key genes commonly identified in both the ILD and COPD groups through WGCNA screening; (**C**) by merging and removing duplicates from the common DEGs in the ILD and COPD groups and the key genes identified through WGCNA, 171 chronic lung disease-related DEGs were obtained. The intersection of these DEGs with ARGs yielded 21 chronic lung disease-related ARGs; (**D**) GO analysis of the 171 DEGs; (**E**) KEEG analysis of the 171 DEGs.

In both the ILD and COPD groups, there were 78 upregulated and 41 downregulated common genes identified through differential analysis, and 57 core genes obtained through WGCNA. By merging these 119 DEGs with the 57 core genes and removing duplicates, a final set of 171 genes associated with chronic lung diseases was established (Figure 4A,B). GO and KEGG analyses of these 171 genes revealed their close association with angiogenesis, vascular development, and immune processes.

Using the GeneCards database, genes related to angiogenesis with a relevance score greater than 3.0 were selected as angiogenesis-related genes (ARGs). By intersecting these ARGs with the previously identified DEGs, we successfully screened out 21 angiogenesis-related genes associated with chronic lung diseases (Figure 4C–E).

### 3.5. Machine Learning for Feature Gene Selection

**Figure 5:**
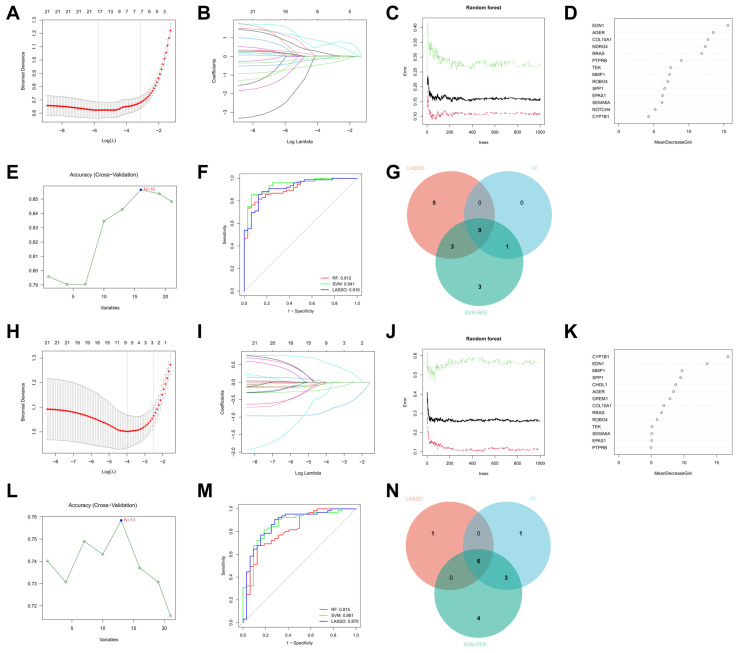
Feature Gene Selection Using LASSO, RF, and SVM-REF. (**A**–**G**) represent the ILD group. (**A**,**B**) LASSO selects 17 feature genes; (**C**,**D**) RF selects the top 10 feature genes; (**E**) SVM-REF selects 16 feature genes; (**F**) ROC curves for feature gene selection by the three machine learning algorithms; (**G**) intersection of feature genes selected by the three machine learning methods yields 9 feature genes. (**H**–**N**) represent the COPD group. (**H**,**I**) LASSO selects seven feature genes; (**J**,**K**) RF selects the top 10 feature genes; (**L**) SVM-REF selects 13 feature genes; (**M**) ROC curves for feature gene selection by the three machine learning algorithms; (**N**) intersection of feature genes selected by the three machine learning methods yields 6 feature genes.

We employed three different machine learning algorithms to screen for feature genes. In the ILD group, 17 genes were selected using the LASSO method, 16 genes were identified through the SVM-REF approach, and the top 10 genes were obtained using the RF method. Ultimately, 9 feature genes were determined (Figure 5A–G). In the COPD group, 7 genes were selected using the LASSO method, 13 genes were identified through the SVM-REF approach, and the top 10 genes were obtained using the RF method. Finally, six feature genes were determined (Figure 5H–N).

### 3.6. Expression and Correlated Features of Target Genes

**Figure 6:**
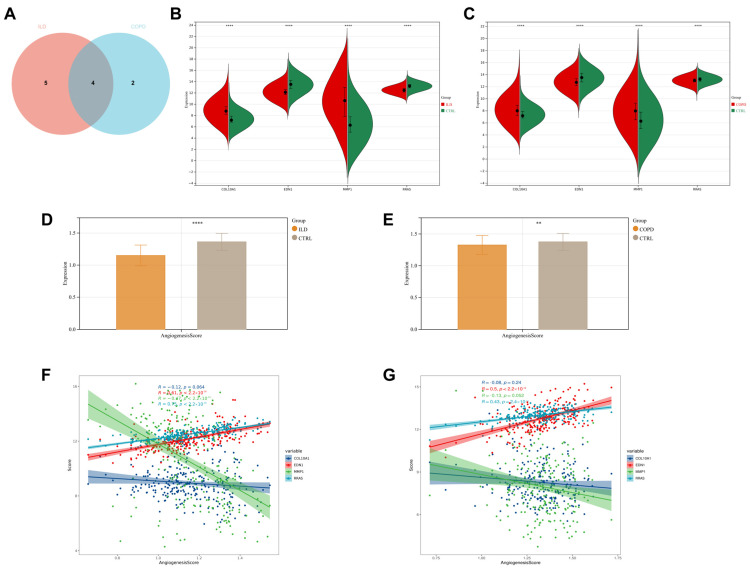
Expression and Correlation Characteristics of Target Genes. (**A**) Among the nine feature genes in the ILD group and the six feature genes in the COPD group, there are four common genes: *COL10A1*, *EDN1*, *MMP1*, and *RRAS*; (**B**) expression levels of the four common feature genes in patients with ILD; (**C**) expression levels of the four common feature genes in patients with COPD; (**D**,**E**) angiogenesis scores; (**F**,**G**) correlation between the four common feature genes and angiogenesis scores. ** *p* < 0.01, **** *p* < 0.0001.

Among the nine feature genes in the ILD group and the six feature genes in the COPD group, there are four common genes: *COL10A1*, *EDN1*, *MMP1*, and *RRAS* (Figure 6A). Specifically, *COL10A1* and *MMP1* are upregulated in the disease groups, while *EDN1* and *RRAS* are downregulated (Figure 6B,C). Next, we assessed their correlation with angiogenesis. Firstly, we calculated angiogenesis scores for the dataset and found that both ILD and COPD groups had lower angiogenesis scores compared to the control group, which is consistent with the results of the GSEA (Figure 6D,E). Then, we examined the correlation between the target genes and the angiogenesis scores. The results indicated that in both ILD and COPD, *COL10A1* and *MMP1* are negatively correlated with angiogenesis, while *EDN1* and *RRAS* are positively correlated with angiogenesis (Figure 6F,G).

### 3.7. Immune Infiltration

**Figure 7:**
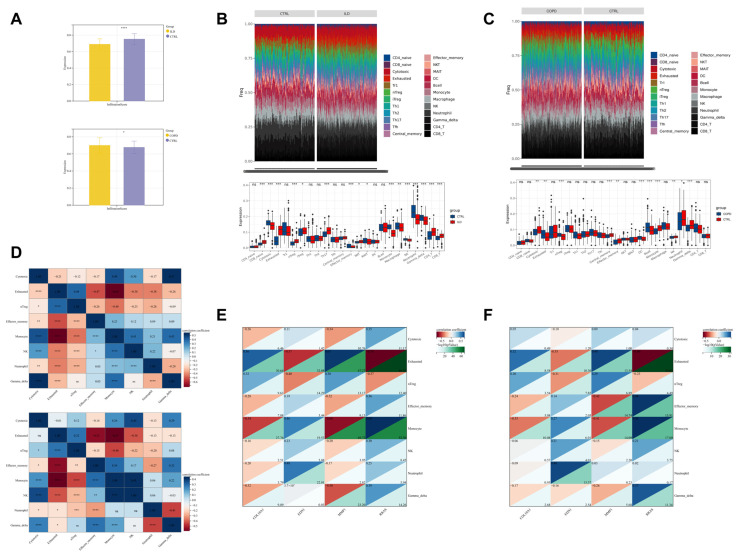
Immune Infiltration Across Different Groups. (**A**) Immune score; (**B**) immune cell infiltration in ILD patients; (**C**) immune cell infiltration in COPD patients; (**D**) correlation of eight types of immune cells—top: ILD, bottom: COPD; (**E**) correlation between the four target genes and immune cells in the ILD group; (**F**) correlation between the four target genes and immune cells in the COPD group. ^ns^ *p* > 0.05, * *p* < 0.05, ** *p* < 0.01, *** *p* < 0.001, **** *p* < 0.0001.

Immune infiltration analysis revealed that the immune scores of ILD patients showed a decreasing trend compared to the control group, while those of COPD patients were higher than the control group (Figure 7A). In ILD patients, the expression levels of cells such as CD8+ naive T cells, exhausted T cells, and natural regulatory T cells (nTreg) were observed to be higher than those in the control group. Conversely, the expression levels of cells such as effector memory T cells, mucosal-associated invariant T cells (MAIT), and monocytes were lower than those in the control group (Figure 7B). In COPD patients, the expression levels of cells such as cytotoxic T cells, exhausted T cells, and nTreg were increased compared to the control group, while the expression levels of cells such as central memory T cells, effector memory T cells, and monocytes showed a decreasing trend (Figure 7C). Notably, the expression trends of exhausted T cells, nTreg, cytotoxic T cells, effector memory T cells, monocytes, natural killer cells, neutrophils, and γδ T cells were consistent between ILD and COPD patients. Correlation analysis further revealed significant correlations among these eight types of immune cells (Figure 7D). In-depth analysis also showed a clear correlation between the target genes and the aforementioned eight types of immune cells (Figure 7E,F).

### 3.8. Clustering Analysis

**Figure 8:**
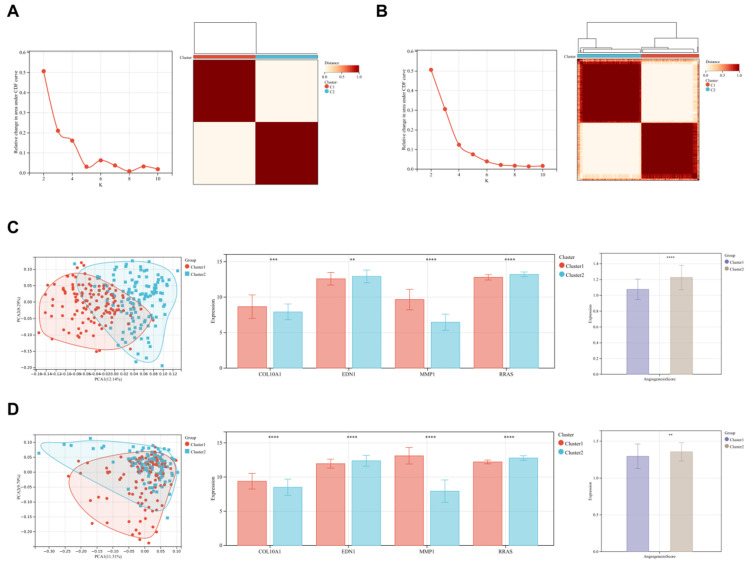
Clustering Analysis Based on the Expression Levels of Four Target Genes. (**A**) Consensus clustering analysis in the ILD group; (**B**) consensus clustering analysis in the COPD group; (**C**) PCA (**left**), expression of target genes (**middle**), and angiogenesis scores (**right**) in two subgroups of ILD patients; (**D**) PCA (**left**), expression of target genes (**middle**), and angiogenesis scores (**right**) in two subgroups of COPD patients. ** *p* < 0.01, *** *p* < 0.001, **** *p* < 0.0001.

We conducted a heterogeneity clustering analysis based on the expression levels of four key genes in the expression profiles of ILD and COPD patients. Principal Component Analysis (PCA) revealed that the expression levels of these four genes can effectively distinguish patients into two subgroups: Cluster 1 (C1) and Cluster 2 (C2). Notably, the expression patterns of the four genes in these two clusters are consistent with the trends observed in the disease states and the healthy control group. Furthermore, Cluster 1 has a lower angiogenesis score compared to Cluster 2, suggesting that Cluster 1 may be more closely associated with the disease state (Figure 8).

### 3.9. Functional Analysis and Immune Status of Different Subgroups

**Figure 9:**
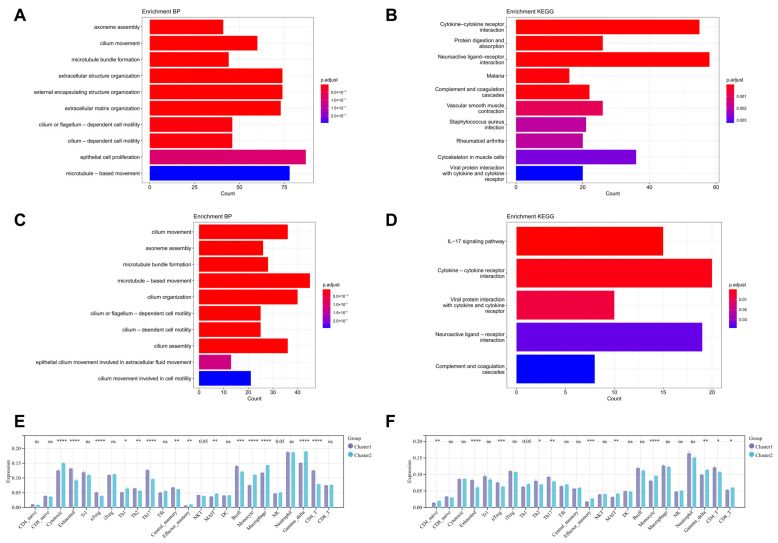
Functional Enrichment and Immune Infiltration Analysis of Different Subgroups. (**A**,**B**) GO and KEGG analysis of differentially expressed genes in the two subgroups within the ILD group; (**C**,**D**) GO and KEGG analysis of differentially expressed genes in the two subgroups within the COPD group; (**E**) immune cell infiltration in the two subgroups within the ILD group; (**F**) immune cell infiltration in the two subgroups within the COPD group. ^ns^ *p* > 0.05,* *p* < 0.05, ** *p* < 0.01, *** *p* < 0.001, **** *p* < 0.0001.

Differential expression analysis was conducted on the data from Cluster 1 and Cluster 2, followed by functional enrichment analysis of the differentially expressed genes. GO analysis revealed that these genes were primarily involved in multiple biological processes related to ciliary motion and function maintenance, epithelial cell proliferation, and extracellular matrix. KEGG analysis identified significant enrichment of these genes in several key biological pathways, including cytokine–cytokine receptor interactions, vascular smooth muscle contraction, complement and coagulation cascades, and the IL-17 signaling pathway (Figure 9A–D). Further examination of immune infiltration status revealed significant differences in the composition of immune cells between Cluster 1 and Cluster 2. Notably, monocytes exhibited a consistent infiltration pattern across the total disease population as well as within each subgroup (Figure 9E,F).

## 4. Discussion

This study identified angiogenesis-related genes in ILD and COPD and delved into the potential mechanisms of angiogenesis in the development and progression of these two diseases. Through systematic analysis, we found that both ILD and COPD exhibited significant changes related to angiogenesis and immune-associated pathways during their pathological processes, suggesting that angiogenesis and the immune system may synergistically contribute to the pathology of ILD and COPD. To further dissect the common pathological mechanisms of these two diseases, we employed differential expression analysis and WGCNA, jointly screening out 171 signature shared genes. Subsequent functional enrichment analysis revealed that these genes were primarily associated with angiogenesis and development, vasculature formation, vascular smooth muscle function, and regulation of the extracellular matrix, and they were closely related to inflammatory responses and immune responses. These findings further support that the interaction between angiogenesis and inflammation may be a central driver in the progression of chronic lung diseases and suggest that these 171 genes may serve as key regulatory factors in the development of chronic lung diseases. Given the important roles of these pathways in the pathological processes of pulmonary artery hypertension and pulmonary fibrosis, we speculate that these shared genes are not only involved in the underlying pathological processes of chronic lung diseases but may also predict the risk of disease progression towards pulmonary artery hypertension and pulmonary fibrosis.

In-depth exploration of immune cells reveals that ILD and COPD exhibit certain differences in immune status, yet multiple immune cell types display similar infiltration characteristics. Notably, monocytes consistently exhibit the same infiltration pattern in both diseases, as well as in the analysis of different subgroups related to angiogenesis-associated genes. This finding strongly suggests that monocytes may play a crucial regulatory role in maintaining angiogenic homeostasis in ILD and COPD. Monocytes are an important source of pulmonary macrophages, and circulating monocytes are recruited to the lungs and differentiate into macrophages to participate in the inflammatory response during lung injury. However, their specific roles in chronic lung diseases remain debated, with different monocyte subsets potentially exerting opposite effects. Previous studies have shown that changes in monocyte counts are associated with the progression of chronic lung diseases, as well as the risk of hospitalization and mortality [27,28]. An earlier study involving 444 COPD patients demonstrated the significance of blood monocytes in identifying individuals at high risk of COPD exacerbations, with patients having a monocyte percentage >10% or <7.4% and an absolute count <0.62 being at higher risk [29]. A study by Ryu et al. [30], which analyzed cell type abundance in RNA-seq data from lung tissues of 1026 COPD and IPF patients, also found that increases in macrophages and classical monocytes were associated with decreased DLCO in IPF and COPD subjects, while lower levels of non-classical monocytes were associated with increased disease severity. In our analysis, monocytes showed a decreasing trend in both ILD and COPD patients, which may be related to the disease state, distribution of monocyte subsets, or functional changes. Future research is needed to delve into the specific mechanisms of different monocyte subsets in chronic lung diseases and their potential regulatory roles in the angiogenic pathway.

In this study, we also identified four key genes: *COL10A1*, *EDN1*, *MMP1*, and *RRAS*. COL10A1 is a pivotal member of the collagen family, which constitutes one of the most essential components of the extracellular matrix (ECM). Multiple studies have revealed that COL10A1 is closely associated with the angiogenic process in tumors, promoting tumor migration, invasion, and epithelial–mesenchymal transition (EMT) [31,32]. For instance, COL10A1 interacts with DDR2 to facilitate cancer cell proliferation and migration, enhancing the migration, invasion, and proliferation of lung adenocarcinoma [33]. In fibrosis, COL10A1 interacts with the integrin subunit β1 (ITGB1), which not only strengthens cell–ECM adhesion but also triggers the TGFβ signaling pathway. As a key pathway regulating cellular proliferation, differentiation, migration, and ECM synthesis, activation of the TGFβ signaling pathway promotes fibroblast activation and proliferation, exacerbating ECM deposition and remodeling, thereby driving the progression of fibrosis [34,35,36]. EDN1 (Endothelin-1) is a pleiotropic bioactive peptide synthesized and secreted by various cells, including endothelial cells. It is a potent vasoconstrictor that exerts vasoconstrictive and mitogenic effects by binding to different receptors on vascular smooth muscle, inducing endothelial dysfunction, inflammation, and angiogenesis, which are crucial mechanisms in the pathogenesis of pulmonary artery hypertension. Additionally, EDN1 drives fibroblast activation, proliferation, and differentiation into myofibroblasts, leading to excessive collagen deposition, a potential risk factor for the development of pulmonary fibrosis [37,38,39,40,41]. MMP1 (Matrix Metalloproteinase 1) is an enzyme ubiquitously present in the human body and belongs to the matrix metalloproteinase (MMPs) family. MMP1 is involved in tissue remodeling, wound healing, and angiogenesis, degrading the ECM to provide necessary space and environment for cell migration, proliferation, and differentiation. Previous studies have shown that elevated MMP1 levels in the elderly may be a risk factor for abnormal pulmonary interstitial changes [42]. In COPD patients, MMP1 expression is also increased, possibly due to its ability to activate inflammatory cells and promote the release of inflammatory factors, thereby exacerbating pulmonary inflammation [43,44]. Furthermore, MMP-1 is elevated in the plasma, serum, and bronchial lavage fluid of IPF patients, and it is significantly overexpressed in IPF compared to normal lung tissue [45]. RRAS (Ras-related protein Rap-1A) is a member of the Ras superfamily and belongs to the small G-protein class. By activating integrins, it enhances adhesion between cells and the ECM, maintaining cellular structural stability and promoting cell migration [46]. RRAS can also activate Akt3, leading to the upregulation of the Notch ligand Jagged1, Notch target genes, and VE-cadherin in adjacent cells, promoting stable interactions between adjacent endothelial cells, which is crucial for maintaining vascular homeostasis [47]. Studies have shown that RRAS can inhibit the secretion of inflammatory mediators such as TNFα and CCL2 by endothelial cells, reduce fibroblast activation, and alleviate pulmonary fibrosis [48].

It is evident that the four genes, *COL10A1*, *EDN1*, *MMP1*, and *RRAS*, are closely linked to immunity, inflammatory responses, and the angiogenic process, likely serving as key regulatory factors in the crosstalk between immunity and angiogenesis. Concurrently, they play crucial roles in the pathogenesis of PAH and pulmonary fibrosis (PF). Therefore, in chronic lung diseases, these genes may be pivotal in maintaining angiogenic homeostasis, regulating the body’s immune status, and preventing the progression of chronic lung diseases towards PAH and PF. Further clustering analysis and immune infiltration analysis have also substantiated our hypothesis. Based on the expression levels of these four genes, the disease group can be distinctly divided into several subgroups, which exhibit significant differences in angiogenic scores and degrees of immune infiltration. Moreover, in the analysis of immune cell infiltration, these four genes demonstrate correlations with the majority of immune cells.

Regarding the merging of datasets, we employed rigorous methods to assess potential batch effects. Apart from not observing significant batch effects, we further validated the uniformity and reliability of the data through clustering analysis, PCA, and other methods. The final gene selection criteria were based on the results of differential expression analysis, WGCNA, and functional enrichment analysis, combined with existing literature and bioinformatics predictions, to comprehensively evaluate the potential roles and value of genes in chronic lung diseases.

This study also has certain shortcomings and limitations. Firstly, this study utilized only one dataset. Although this dataset comprises a large number of patient specimens and clinical data, the analysis results may have some limitations. Future studies should validate and delve deeper into the findings using more datasets and clinical samples. Secondly, the expression profiles in this dataset were generated using two platforms. Although we did not observe significant batch effects when merging the datasets, there may be some discrepancies between different platforms. We have assessed potential batch effects using rigorous methods and taken them into account in our analyses. Lastly, we relied solely on bioinformatics for data analysis. Although we validated our results using methods such as clustering analysis and immune infiltration, there are still certain limitations. Future studies may require clinical and basic experiments to further validate our findings and explore the mechanisms of action of the target genes and monocytes in chronic lung diseases.

In summary, we have explored the crucial roles of angiogenesis and immunity in the onset and progression of ILD and COPD, and we identified *COL10A1*, *EDN1*, *MMP1*, and *RRAS* as potential novel therapeutic targets for chronic lung diseases. This finding provides important scientific evidence for a deeper understanding of the molecular mechanisms underlying chronic lung diseases and the exploration of novel therapeutic strategies, which may slow the further deterioration of patients’ conditions, prevent the development of pulmonary fibrosis and pulmonary artery hypertension, and, thereby, improve patients’ quality of life. Future research will further validate the mechanisms of action of these genes in chronic lung diseases and explore their potential applications in clinical diagnosis and treatment.

## Data Availability

All data can be obtained in the public database.
